# Strontium Modified Calcium Sulfate Hemihydrate Scaffold Incorporating Ginsenoside Rg1/Gelatin Microspheres for Bone Regeneration

**DOI:** 10.3389/fbioe.2020.00888

**Published:** 2020-08-18

**Authors:** Peng Luo, Lan Yu, Qiang Lin, Changde Wang, Dazhi Yang, Shuo Tang

**Affiliations:** ^1^Department of Orthopaedics, Huazhong University of Science and Technology Union Shenzhen Hospital (Nanshan Hospital), Shenzhen, China; ^2^Department of Orthopaedics, The 6th Affiliated Hospital of Shenzhen University Health Science Center, Shenzhen, China; ^3^Department of Laboratory, Huazhong University of Science and Technology Union Shenzhen Hospital (Nanshan Hospital), Shenzhen, China; ^4^Department of Orthopaedics, Guangdong Hospital of Traditional Chinese Medicine, Guangzhou, China; ^5^Department of Geriatric Orthopeadics, Shenzhen Pingle Orthopaedic Hospital, Shenzhen, China; ^6^Department of Orthopaedics, The Eighth Affiliated Hospital, Sun Yat-sen University, Shenzhen, China

**Keywords:** Ginsenoside Rg1 (Rg1), gelatin microspheres, strontium (Sr), calcium sulfate hemihydrate (α-CaS), bone defect

## Abstract

The aim of this study was to prepare a promising biomaterial for bone tissue repair and regeneration. The Strontium – calcium sulfate hemihydrate (Sr-α-CaS) scaffold incorporating gelatin microspheres (GMs) encapsulated with Ginsenoside Rg1 (Rg1) was designed. The scaffolds of Rg1/GMs/Sr-α-CaS showed sustained release of Rg1, good biocompatibility and ability of promoting osteogenic differentiation and angiogenesis *in vitro*. The scaffolds were implanted into animal model of cranial bone defect to characterize bone tissue repair and regeneration *in vivo*. From the images of Micro-CT, it was obvious that the most bone tissue was formed in Rg1/GMs/Sr-α-CaS group in 12 weeks. New bone structure, collagen and mineralization were analyzed with staining of HE, Masson and Safranin O-Fast green and showed good distribution. The expression of osteocalcin of Rg1/GMs/Sr-α-CaS indicated new bone formation in defect site. The results revealed that synergy of Rg1 and Sr showed the best effect of bone repair and regeneration, which provided a new candidate for bone defect repair in clinic.

## Introduction

Large bone defect caused by trauma and tumor resection is still a severe problem in clinic for the limited self-repair capability and absence of transplant. Tissue engineering provides a feasible method for bone repair and regeneration (Porter et al., 2009). Compatible mechanical strength and good biocompatibility are especially important for ideal substitute of bone tissue. Inorganic biomaterials of hydroxyapatite, β-Tricalcium phosphate, and calcium sulfate has been reported widely (Gotterbarm et al., 2014; Ramesh et al., 2018; Meng et al., 2019). Calcium sulfate has been used as a bone defect filling and repairing material for over a 100 years. It has good biocompatibility and can be degraded and absorbed into the body without immune reaction (Kutkut and Andreana, 2010). Commercial medical calcium sulfate products such as Osteoset (Wright, United States) are used into clinic, which change CaSO_4_⋅2H_2_O into CaSO_4_⋅0.5H_2_O and can be degraded within 6–8 weeks *in vivo* (Winn and Hollinger, 2000). α-CaSO_4_⋅0.5H_2_O loading bone morphogenetic proteins-2 (BMP-2) peptide was reported to repair a critical defect in the femoral condyle of rabbit (Liu et al., 2015b). The rapid solidification of α-CaSO_4_⋅0.5H_2_O make it a candidate as bone cement. α-CaSO_4_⋅0.5H_2_O was mixed with bioactive glasses and the mixture had potential used as the substitute of bone implant (Zheng et al., 2018b).

Strontium (Sr) is an alkaline earth metal element and plays an important physiological function in human body. Strontium ranelate is used to treat severe osteoporosis in postmenopausal women authorized by the European Union (Barenholdt et al., 2009). Sr is also used widely in bone tissue engineering. Sr can substitute Calcium (Ca) for their similar ion diameter, and when the concentration of Sr > 0.2 mol L^–1^, it would induce partial conversion of Calcium sulfate dihydrate to hemihydrate (Feldmann and George, 2013). Sr also has bone-seeking properties, and it can replace Ca to strength mechanical behaviors (Jimenez et al., 2019). The cement of Sr-containing α-calcium sulfate hemihydrate was reported with enhanced osteoblastic differentiation, new bone and new blood vessel formation in critical-sized calvarial defects *in vivo* (Yang et al., 2017). In our previous study, Sr substituted hydroxyapatite/silk fibroin scaffold loading BMP-2 increased bone mineral density (BMD) and improved new bone regeneration (Yan et al., 2018). For mesenchymal stem cells (MSCs), it was found that Sr activated Wnt/Catenin signaling to promote osteogenic differentiation *in vitro* and *vivo* (Yang et al., 2011). Sr also enhanced osteoblast activity and inhibited osteoclast activity (Wornham et al., 2014).

Ginsenoside Rg1 extracted from dry root of Panax ginseng C. A. Mey has many physiological functions for the antioxidant and anti-inflammatory properties (Gao et al., 2017b; Ghaeminia et al., 2018). For bone repair and regeneration, Rg1 was demonstrated to have protective effects on BMSCs apoptosis by activating mir-494-3p. ROCK-1. Bcl-2 signaling pathway in male rat (Zheng et al., 2018a). Rg1 was also reported to promote osteogenic differentiation with activated GR/BMP-2 signaling pathway *in vitro* and enhance bone calcification to accelerate the fracture healing *in vivo* (Gu et al., 2016). For osteonecrosis lesion, Rg1 stimulated capillary vessel formation and increased BMD (Heng et al., 2019). Furthermore, Rg1-loaded alginate-chitosan microspheres were prepared, and the controllable release of Rg1 promoted proliferation and differentiation and suppressed apoptosis of bone marrow stromal cells (hBMSCs) (Guo et al., 2017).

In this study, we designed a scaffold of Rg1/GMs/Sr-α-CaS to repair calvarial bone injury, which has not been reported before. Sr-α-CaS provided mechanical support, and Rg1 and Sr were released to repair bone defect. To avoid the burst release of drug, we introduced gelatin microspheres as drug carriers and prepared Rg1/GMs. We evaluated the structure and composition, biocompatibility, and the ability to induce osteogenic differentiation *in vitro* and tissue repair and regeneration monitored by Micro-CT *in vivo*. The expression of osteocalcin (OCN) and osteogenesis were evaluated.

## Materials and Methods

Ginsenoside Rg1 (>98%, Aladdin, China); Gelatin (Porcine skin, Type A, Sigma-Aldrich, United States); Span 80 (Aladdin, China); Olive oil (Aladdin, China); SrCl_2_⋅6H_2_O (Aladdin, China); Gibco MEM α, Nucleosides (Thermo Fisher Scientific, United States); Fetal bovine serum (FBS, Sigma-Aldrich, United States); Anti-Osteocalcin (Anti-OCN, Santa Cruz Biotechnology, United States); CCK-8 Kit (Beyotime, China); ALP Kit (Beyotime, China); RNeasy Mini Kit (Qiagen, United States); cDNA synthesis Kit (Beyotime, China); Bulge-LoopTM miRNA qRT-PCR kit (Ribobio, Guangzhou, China); Hematoxylin and Eosin Staining Kit (Beyotime, China); Masson’s Trichrome Stain Kit (MKbio, Shanghai, China); and Safranin O-Fast green Stain Kit (Servicebio, Wuhan, China).

### Cell Culture

MC3T3-E1 cells (Biowit Biotech, Shenzhen, China) were cultured in MEMα culture medium supplemented with 10% FBS and 1% Penicillin-Streptomycin at 37°C, 5% CO_2_ humidified atmosphere. The medium was changed every 2 days for further experiment.

### Preparation of Rg1 Loaded Gelatin Microspheres (Rg1/GMs)

Gelatin microspheres (GMs) were prepared with an emulsion solvent evaporation method with a slight modification (Yang et al., 2009). 0.1 g sorbitol oleate (Span-80) was added to 100 mL olive oil, heating in a water bath at 60°C for 0.5 h at 400 rpm. After mixing evenly, add 10 mL gelatin solution of 10 wt% drop by drop. After stirring for 3 h, transfer the mixed solution to the ice bath and keep the rotation speed for 30 min. Then, 0.1 mL of 25 wt% aqueous solution of glutaraldehyde was added. After 2 h stirring, add 30 mL acetone at 4°C and stir for 30 min. The product was placed into 10 mL acetone for further curing for about 24 h at 4°C. Then, the microspheres were gained and soaked into 1 mol/L aminoacetic acid for 30 min and washed alternately by ethanol and isopropyl alcohol for three times. Finally, freeze-drying to obtain GMs. Rg1/GMs were prepared similarly. 10 mg Rg1 was dispersed into 10 mL gelatin solution of 10 wt%. Then, the mixture was added in olive oil with Span-80, and repeat the methods as above. Rg1 was loaded into GMs to form Rg1/GMs. The microspheres with different mass ratio of Rg1 and GMs were prepared, and cell proliferation of MC3T3-E1 was used to evaluate the best dosage of Rg1.

### Preparation of Rg1/GMs/Sr-α-CaS Scaffold

α-CaSO_4_⋅0.5H_2_O (α-CaS) calcium sulfate hemihydrate was synthesized first (Li et al., 2014). The composite crystallizer was prepared using sodium citrate, aluminum sulfate and succinic acid with a mass ratio of 1:1:1. Solution of CaCl_2_ and K_2_SO_4_ was evenly stirred with molar mass ratio of 1:1 in a sealed hydrothermal reaction vessel, and then adding composite crystallizer into vessel with dosage at 5% of the total mass of CaCl_2_ and K_2_SO4. Dilute HCl was used to keep pH 5. Hydrothermal reaction was conducted for 4 h at 80°C, 300 r/min. The product was washed with hot water for three times and soaked in absolute ethanol to stop the reaction. After filtering, dry product in a vacuum oven for 4 h and at 105°C and get the final product of α-CaS. α-CaS was fully ground and sealed for next use. Sr-α-CaS was prepared as above. SrCl_2_⋅6H_2_O and CaCl_2_ were dissolved and mixed with a molar ratio of 1:6. Then add K_2_SO_4_ solution and keep the molar ratio at 1: 1 (m_Sr_^2+^& Ca^2+^: m_SO4_^2–^). Repeat the above steps to get Sr-α-CaS. Rg1/GMs and Sr-α-CaS were fully mixed up with a mass ratio of 1: 100 by a planetary mixer (Nova, Shanghai, China) at 50–200 r/min. The homogeneous powder was pressed into a sheet-like scaffold with a tableting machine (Lvyi, Shanghai, China) under 10–20 MPa.

### Characterization of Rg1/GMs/Sr-α-CaS Scaffold

Morphology of gelatin microspheres was measured by scanning electron microscope (SEM, Philips XL-30; Philips, Netherlands). Crystal structure of α-CaS and Sr-α-CaS was tested by X-ray diffractometer (XRD, Gemini S Ultra, Oxford Diffraction Ltd., Japan). The infrared (IR) spectra were obtained by Fourier transform infrared spectroscope (FTIR, VERTEX70, Bruker, Germany). The size and shape of α-CaS were determined by Transmission electron microscopy (TEM, Philips Tecnai-10; Philips, Netherlands) and quantitative analysis of elements was performed by Energy Dispersive Spectrometer (TEM-EDS). Composition was detected by Thermogravimetric analyzer (TG, TG 209; NETZSCH, Germany).

### Loading Rate and Release of Rg1 *in vitro*

The quantification of Rg1 was detected by High performance liquid chromatography (HPLC, UltiMate 3000; Thermo Fisher Scientific, United States). The mobile phase was composed with acetonitrile (mobile phase) and 0.05% Na_2_HPO_4_ (PB, pH: 7, mobile phase B). The detection wavelength was selected at 203 nm. The standard Rg1 solution of 1 mg/mL was prepared and diluted into different concentrations. The HPLC assay was processed. Draw the standard curve of Rg1 and calculate the loading rate of Rg1/GMs. Scaffolds of Rg1/Sr-α-CaS and Rg1/GMs/Sr-α-CaS and 10 mg microspheres of Rg1/GMs were soaked into 10 mL PBS with pH 7.4 at 37°C. At the set time, 0.5 mL supernatant was collected and replaced by 0.5 mL fresh PBS. The collected supernatant was stored at –20°C. After all the samples were collected, the HPLC assay was carries out at 203 nm as above. The cumulative release of Rg1 was calculated via standard curve line. All experiments repeated in triplicate for each time interval and draw the cumulative release curve.

### Cytotoxicity Assay *in vitro*

Leaching solution of each scaffold of Sr-α-CaS, Rg1/Sr-α-CaS and Rg1/GMs/Sr-α-CaS was prepared according to international standard. In brief, scaffolds were soaked into ethanol of 75% and conducted UV radiation for 30 min, respectively. Then, wash scaffolds with PBS for 2–3 times. Add medium with ten times the mass of scaffolds and incubate for 24 h at 37°C. MC3T3-E1 cells in logarithmic growth phase were digested and adjusted to 5 × 10^4^/ mL. 100 μL cell suspension was added to 96-well plates. After growth for 24 h, replace the medium with leaching solution and continue to culture cells for 1, 4, 7, and 10 days. CCK-8 Kit was used to evaluate cell viability. Add CCK-8 reagent with a volume ratio of 1:9 (V_medium_: V_CCK–8_ = 1:9) and incubate for 30 min according to manufacturer’s instruction. Test the absorbance wavelength at 450 nm of each well using a microplate reader (MM-58938-00, Molecular Devices, United States) and calculate the cell viability.

### Alkaline Phosphatase (ALP) *in vitro*

The osteogenic differentiation of MC3T3-E1 was evaluated with ALP activity test, which was a distinct feature of osteoblast differentiation. MC3T3-E1 cells were digested and adjusted to 3 × 10^4^/mL, and 500 μL cell suspension was adding to 24-well plates. After incubating for 24 h, replace medium with 500 μL leaching solution. While culturing for 1, 7, 14, and 21 days, wash cells with PBS for three times and add 500 μL cell lysates. Cells were disrupted using an ultrasonic cell disrupter (Fisher Scientific 50, Thermo Fisher Scientific, United States) at 4°C. Centrifugate and collect supernatants, add 500 μL ALP substrate reaction solution. The reaction was carried out for 30 min at 37°C. 500 μL of 0.1 M NaOH was added to terminate the reaction. The UV absorption at 405 nm was then determined using ultraviolet-visible spectrophotometer (UV-2550, Shimadzu, Japan). ALP activity was calculated according to instructions. Each group of scaffolds at each time point was tested at least three times in parallel.

### VEGF Expression *in vitro*

MC3T3-E1 cells were cultured with leaching solution in 24-well plates as above for 1, 4, 7, and 21 days. The cells were collected and total RNA was extracted with RNeasy Mini Kit. Reverse transcription of total RNA to obtain cDNA according manufacture instruction. cDNAs and equal amounts of forward and reverse primers were added into a SYBR reaction mixture. The qPCR conditions were 2 min at 50°C, 10 min at 95°C, 50 cycles at 95°C for 15 s, and 1 min at 60°C. miRNA qRT-PCR was detected by the Bulge-LoopTM miRNA qRT-PCR kit. VEGF forward primers: 5′-ACAGAAGGGGAGCAGAAAGCCCAT-3′; Reverse primer: 5′-CGCTCTGACCAAGGCTCACAGT-3′; GAPDH forward primer: 5′-AGGTCGGTGTCAACGGATTT and reverse primer: 3′-CCTTCCACGATGCCAAAGTT.

### Animal Model of Cranial Bone Defect

All animal experiments were strictly carried out in accordance with the regulations of the Animal Research Center of Sun Yat-sen University. 18 SD rats (200–250 g) were divided into three groups (*n* = 6): Sr-α-CaS, Rg1/Sr-α-CaS and Rg1/GMs/Sr-α-CaS. The scaffold (diameter: 8 mm, weight: 183.3 mg) implantation into calvarial defect was shown in Supplementary Figure S5. Before the operation, sodium pentobarbital solution of 1% was intraperitoneally injected into SD rats at a dose of 30 mg/kg. Remove the hair of the rat brain, and cut a 3 cm long scalp in the middle of the rat’s head. At the same time, the subcutaneous tissue on both sides was peeled off. After removing the periosteum, a calvarial defect model with a diameter of 6 mm was constructed using a dental drill in the middle of the rat’s parietal bone. Take care during the operation to maintain the integrity of the dura mater. The sterilized scaffolds were sequentially implanted into the calvarial defects as shown in Supplementary Figure S5C, and the periosteum and scalp were sutured (Supplementary Figure S5D). After 4, 8, and 12 weeks of implantation, the rats were sacrificed and the skulls were dissected and placed in a 4% paraformaldehyde solution for subsequent histological characterization.

### Micro-CT *in vivo*

After 4, 8, and 12 weeks postoperatively, the rats were anesthetized, and then the defect was analyzed using the Micro-CT imaging system (BRUKER Micro-CT Sky Scan 1176, Bruker, United States). The parameters of the device were set: the layer thickness: 48 μm; the spacing between the layers: 48 μm; the pixel size: 48 μm and it was also set to high voltage mode. The bone volume and bone density of regenerated bone tissue were calculated using the software provided by Micro-CT.

### Histological Analysis

After 4, 8, and 12 weeks, the removed samples were collected and soaked into 4% solution of paraformaldehyde at room temperature for 24 h. Subsequently, the fixed samples were immersed in 10% EDTA solution for decalcification of about 45 days, and the decalcification solution was changed every 3 days until the bone tissue needle can pass smoothly. Then, different concentrations of ethanol solution were used for dehydration. The samples were subjected to lateral paraffin embedding, and sliced vertically with 5 μm thickness. Finally, staining of hematoxylin-eosin (H&E), Masson’s trichrome and Safranin/solid green were performed to characterize the bone tissue repair and regeneration.

### Immunohistochemistry

Osteocalcin was a protein formed by osteoblasts to constitute new bone. The antibody of anti-OCN was used to evaluate the regeneration of bone tissue. The samples were prepared into sections as above. Sections were incubated with anti-OCN primary antibody (1: 200) overnight at 4°C. Then, add a secondary antibody (1: 500) to incubate for 2 h at room temperature. Diaminobenzidine (DAB) coloring agent was to perform protein distribution and observe OCN expression with light microscope (Olympus IX71, Japan).

### Statistical Analysis

The data of this study were averaged ± standard deviation (SD) of statistical data using SPSS software (version 16.0; SPSS, Chicago, IL, United States). At least three parallel samples (*n* = 3) were set for all experiments. ^∗^ indicates *p* < 0.05, ^∗∗^ indicates *p* < 0.01, and the smaller the *p*-value, the more the corresponding ^∗^ is, the difference of *p* < 0.05 is considered to be statistically significant.

## Results

### Characteristics of Scaffolds

**FIGURE 1:**
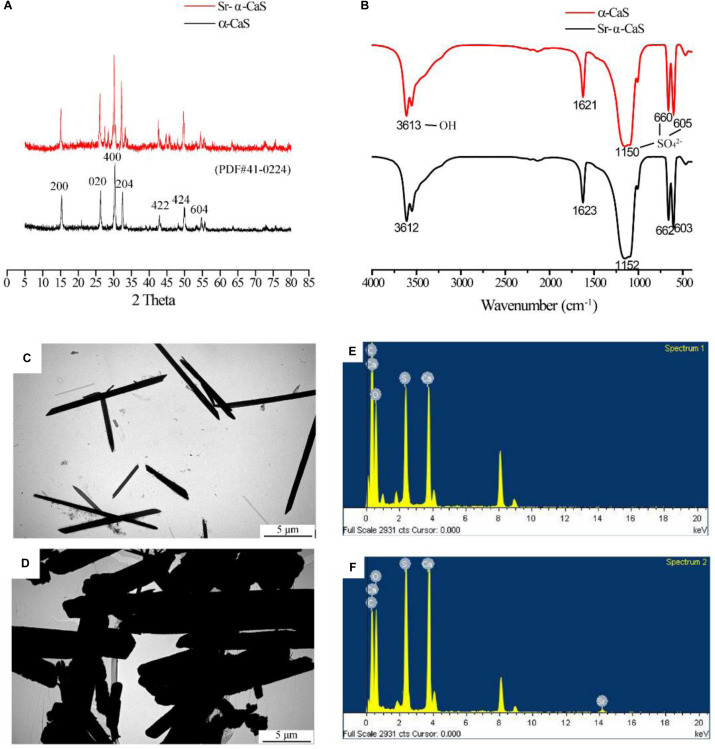
XRD pattern **(A)**, FTIR spectrum **(B)** and TEM image of α-CaS **(C)** and Sr-α-CaS **(D)**, EDS of α-CaS **(E)** and Sr-α-CaS **(F)**.

**FIGURE 2:**
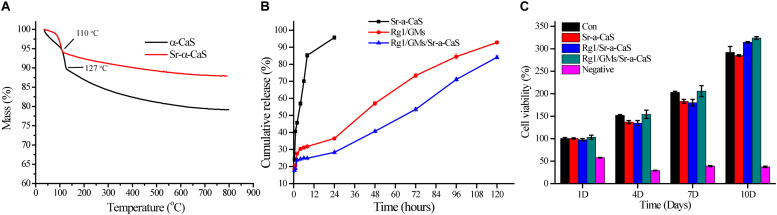
TG **(A)** of α-CaS and Sr-α-CaS; cumulative release curve **(B)** of Ginsenoside Rg1 of Rg1/Sr-α-CaS, Rg1/GMs and Rg1/GMs/Sr-α-CaS; MC3T3-E1 cell viability **(C)** on different scaffolds.

The XRD diffraction patterns were shown in Figure 1A, the diffraction peaks of α-CaS at 15.1° (200), 26.2° (020), 30.5° (400), 32.6° (204), 43.0° (422), 50.1° (424), and 54.8° (604) were consistent with standard α-CaSO_4_⋅0.5H_2_O (PDF#41-0224). Sr-α-CaS had similar characteristic peaks with α-CaS, and diffraction peaks of Sr^2+^ presented at 27.5° and 28.5° (Li et al., 2014). FTIR spectrum of α-CaS was in Figure 1B. Characteristic absorption peaks at 1150, 660, and 605 cm^–1^ presented asymmetric stretching vibration and bending vibration of SO_4_^2–^ and characteristic peak at 3613 cm^–1^ was vibration of O-H. The results of XRD and FTIR of α-CaS indicated α-CaSO_4_⋅0.5H_2_O was successfully synthesized, and with the incorporation of Sr, the crystal structure of α-CaS was not significantly affected. TEM images was showed in Figures 1C,D. Whisker of α-CaS can be observed while Sr-α-CaS appeared larger particle and size thicker rod, which may be caused by the different growth rate of Sr^2+^ in the length direction and diameter direction of whisker surface. Elemental analysis from EDS in Figures 1E,F and Table 1 showed the content of Sr was 16.1%. The composition of Sr-α-CaS was also tested by TG in Figure 2A. As temperature rise, both mass of α-CaS and Sr-α-CaS declined, and content of Sr was about 8.7%. Calcium sulfate hemihydrate containing 16.1% Sr (Sr-α-CaS) was prepared successfully. The SEM of gelatin microspheres was in Supplementary Figure S1, and the average diameter was 9.24 ± 0.87 μm, surface area was 268.09 ± 50.48 μm^2^. The average diameter of pores in microspheres was also measured, which was 0.59 ± 0.06 μm. The SEM images of scaffolds were in Supplementary Figure S2. Compared with Sr-α-CaS, Rg1/GMs/Sr-α-CaS scaffolds showed rougher surface and round gelatin microspheres can be observed. Cell proliferation of MC3T3-E1 cells cultured with different ratio of Rg1/GMs was shown in Supplementary Figure S3. While Rg1/GMs ratio was 10:1, the highest proliferation rate can be obtained.

**TABLE 1:** The element weight of different α-CaS and Sr-α-CaS.

	**C**	**O**	**S**	**Sr**	**Ca**	**Sr/(Sr+Ca)**
	
	**Weight (%)**	**Weight (%)**	**Weight (%)**	**Weight (%)**	**Weight (%)**	**Weight (%)**
α-CaS	49.29	21.12	13.80	0	15.79	0
10% Sr-α-CaS	30.82	24.26	20.38	16.1	22.93	6.56

### Loading Rate and Release of Rg1 *in vitro*

The standard curve of Rg1 was in Supplementary Figure S4, regression equation was *Y* = 0.00426*X* − 0.0002835, *R*^2^ = 0.9993, linear range was 3.125–50.000 mg/L, which showed good correlation in the linear range. The loading rate of Rg1/GMs was obtained by calculating the peak area in chromatograms, and it was 2.51% (w/w). Cumulative release curve of Rg1 was in Figure 2B, which was calculated using the standard curve. In first 36 h, Rg1 was almost completely released from Rg1/Sr-α-CaS. However, for Rg1/GMs and Rg1/GMs/Sr-α-CaS, release rate decreased significantly and Rg1 showed sustained release without drug burst. Compared with Rg1/GMs, release rate of Rg1/GMs/Sr-α-CaS was always slower, and cumulative release of Rg1/GMs was 92% while it was 85% in Rg1/GMs/Sr-α-CaS within 120 h. Although the scaffolds of Sr-α-CaS had good degradability, GMs can be used as drug carriers to maintain sustained release of Rg1, which caused the above results. It demonstrated that Rg1/GMs/Sr-α-CaS can provide Rg1 stably to repair bone defect *in vivo*.

### Cytotoxicity *in vitro*

To evaluate biocompatibility of scaffolds, cytotoxicity was conducted and 0.5wt% phenol was used as negative group. After culturing MC3T3-E1 cells for 1, 4, 7, and 10 days with leaching solution, cell viability was measured by CCK-8 assay in Figure 2C. Compared with control group, all the scaffolds showed good biocompatibility, and cell viability was more than 90%. The results demonstrated Sr-α-CaS was safe and incorporation of Sr did not affect the biocompatibility of scaffolds. What’s more, Rg1/GMs/Sr-α-CaS showed the highest cell viability and it may be caused by slight release of Rg1.

### ALP *in vitro*

**FIGURE 3:**
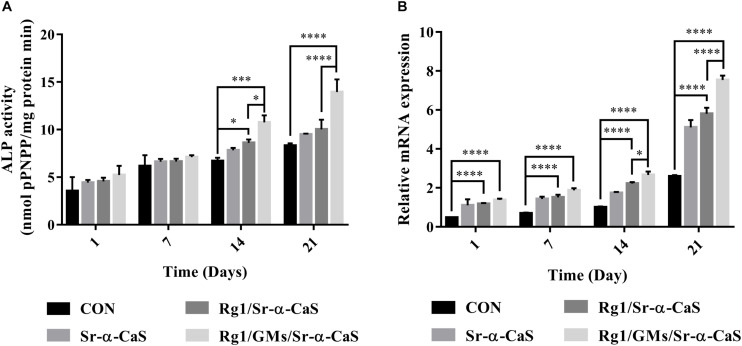
The ALP **(A)** and VEGF **(B)** assay of MC3T3-E1 cultured on different scaffolds. ^∗^*p* < 0.05, ^∗∗^*p* < 0.01, ^∗∗∗^*p* < 0.001, ^****^p < 0.0001.

The expression of ALP in Figure 3A indicated the osteogenic differentiation of MC3T3-E1. After culturing for 1, 7, 14, and 21 days, ALP activity gradually increased with time, and all scaffolds presented higher ALP activity than control group, which indicated each scaffold can provide support for osteogenic differentiation of MC3T3-E1. Scaffolds with Sr and Rg1 showed higher ALP expression in 14 and 21 days. Therefore, synergistic effect of Rg1 and Sr prompted osteogenic differentiation. However, compared with Rg1/Sr-α-CaS group, ALP activity of Rg1/GMs/Sr-α-CaS showed significant increase at 14 and 21 days. Due to sustained release of Rg1 from GMs, concentration of Rg1 in leaching solution of Rg1/Sr-α-CaS was higher than Rg1/GMs/Sr-α-CaS group, which was consistent with the result of cumulative release curve. Therefore, it was inferred that low concentration of Rg1 was more conducive to osteogenic differentiation.

### VEGF Expression *in vitro*

VEGF expression was related to blood vessel formation, and it was measured by RT-qPCR at 1, 7, 14, and 21 days. In Figure 3B, all the scaffold groups showed significant increase in VEGF expression. Scaffolds with Sr and Rg1 also showed higher expression of VEGF than other group in 14 and 21 days. But compared with Rg1/Sr-α-CaS, VEGF expression of Rg1/GMs/Sr-α-CaS was increased obviously, which was consistent with results of ALP expression. It was also inferred that low concentration of Rg1 can effectively promote vascularization.

### Micro-CT

**FIGURE 4:**
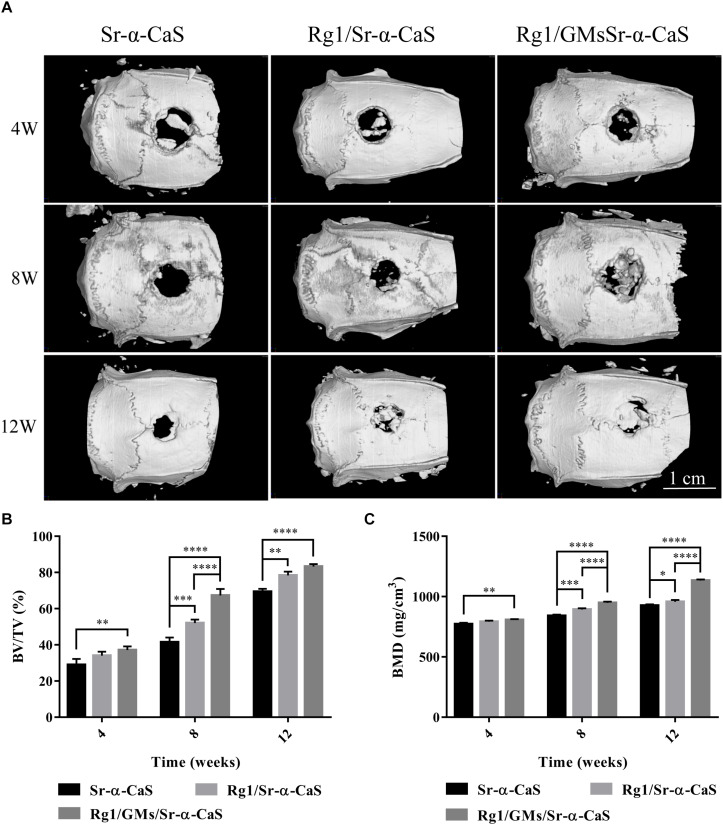
Micro-CT images **(A)** of different scaffolds in rat calvarial defects; Micro-CT analysis of BT/TV **(B)** and BMD **(C)** in the rat calvarial defects. ^∗^*p* < 0.05, ^∗∗^*p* < 0.01, ^∗∗∗^*p* < 0.001, ^****^p < 0.0001.

Scaffolds of Sr-α-CaS, Rg1/Sr-α-CaS, and Rg1/GMs/Sr-α-CaS were implanted into skull defect. The repair and regeneration in defect site were evaluated by Micro-CT in Figure 4A. It was obvious that more bone tissue was observed in scaffolds with Sr and Rg1 for 12 weeks. Compared with Rg1/Sr-α-CaS group, more bone tissue was formed in Rg1/GMs/Sr-α-CaS group for 8 weeks, thus sustained Rg1 and Sr appeared the highest efficiency of cranial bone repair. New bone volume and BMD was calculated in Figures 4B,C. The volume of new bone increased with time for each scaffold, and Rg1/GMs/Sr-α-CaS group always showed the most bone volume. At 12 week, the BV/TV of Rg1/GMs/Sr-α-CaS rise up to 83% while it was only 78% in Rg1/Sr-α-CaS and 69% in Sr-α-CaS. BMD also increased with time. Scaffolds with Rg1 showed higher BMD than Sr-α-CaS, and Rg1/GMs/Sr-α-CaS group always had the highest BMD *in vivo*. It revealed that Rg1 and Sr promoted new bone formation with enhanced bone volume and BMD.

### Histology

**FIGURE 5:**
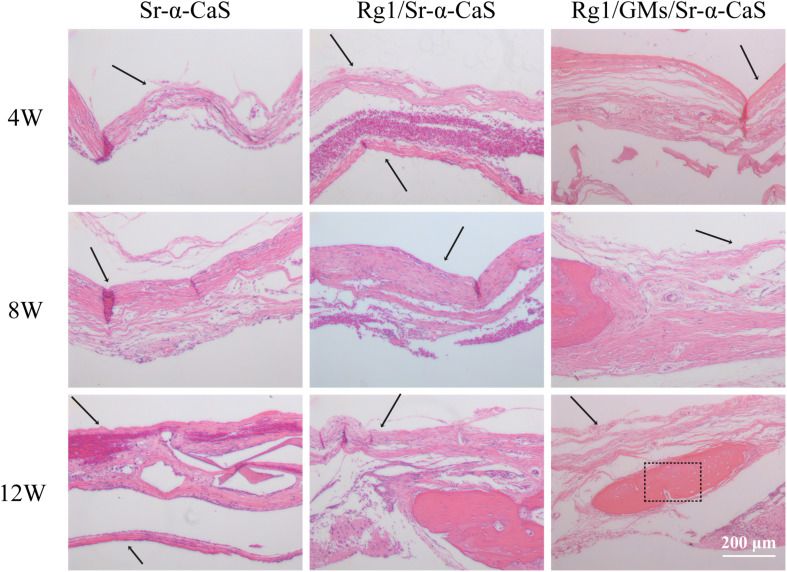
Hematoxylin and eosin (H&E) staining photomicrographs of Sr-α-CaS, Rg1/Sr-α-CaS and Rg1/GMS/Sr-α-CaS groups (Magnification = 100×, black arrow: trabeculae, black rectangle: bone tissue).

HE staining of bone tissues in defect site for 4, 8, and 12 weeks of was exhibited in Figure 5. After 4 week of implantation, no obvious inflammatory response was found in all groups and a small amount of bone trabecula (black arrows) was observed in Rg1/GMs/Sr-α-CaS group. After 8 week, bone trabecula and fibrous tissue in defect site were found. For 12 week, the trabecular bone became thicker and a large number of new bone formation was observed in all groups, and the morphology of newborn bone tissue (black rectangle) in Rg1/GMs/Sr-α-CaS was similar with normal tissue.

**FIGURE 6:**
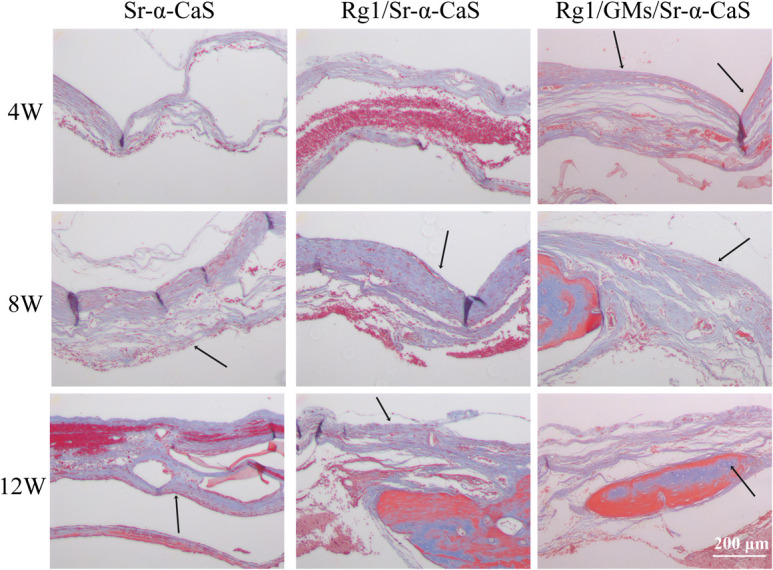
Masson staining of Sr-α-CaS, Rg1/Sr-α-CaS and Rg1/GMS/Sr-α-CaS (Magnification = 100×, black arrow: collagen fibers).

**FIGURE 7:**
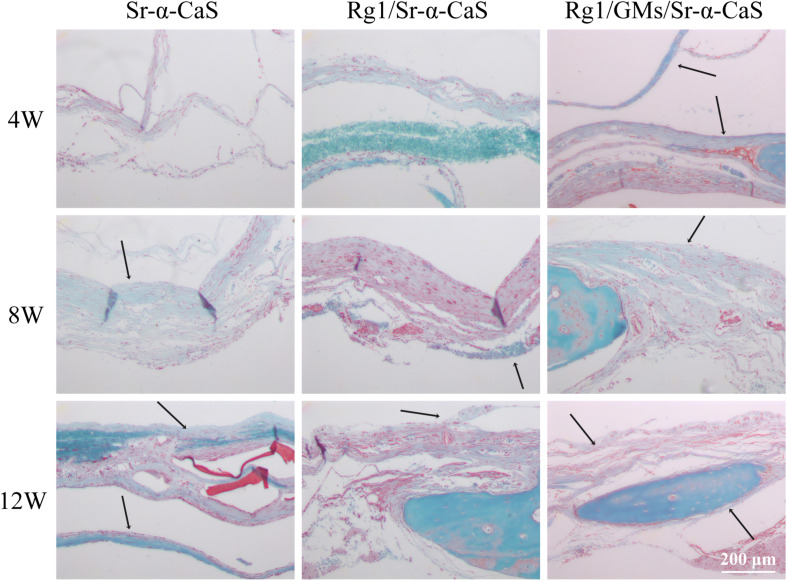
Safranin O and Fast Green staining photomicrographs of calvarial defect sites treated with Sr-α-CaS, Gim/Sr-α-CaS and Gim/GMS/Sr-α-CaS (Magnification = 100×, black arrow: bone tissue).

The formation of collagen fibers in 4, 8, and 12 weeks showed in Figure 6 and Supplementary Figure S6A. From the result of Masson staining in 4 week, there was no significant difference between Sr-α-CaS and Rg1/Sr-α-CaS, but for Rg1/GMs/Sr-α-CaS group, fiber tissue (black arrows) can be observed. After 8 week of transplantation, filamentous collagen fibers formed at the defect for all groups. After 12 week, the new tissue of Rg1/GMs/Sr-α-CaS was the most closet with normal bone tissue and the most collagen fiber was observed. The results of Safranin O-Fast green Staining was in Figure 7 and Supplementary Figure S6B. Green newborn bone tissue (black arrows) was not apparent in 4 weeks. After 8 weeks, fibrous new bone trabecular was formed in all groups. After 12 week of transplantation, a large number of new bones was observed, and bone in Rg1/GMs/Sr-α-CaS also showed closet structure with normal bone tissue, which was consistent with the staining results of HE and Masson. Therefore, scaffold of Rg1/GMs/Sr-α-CaS showed good cranial bone repair, which accelerated the formation of collagen and bone.

### Immunohistochemistry

**FIGURE 8:**
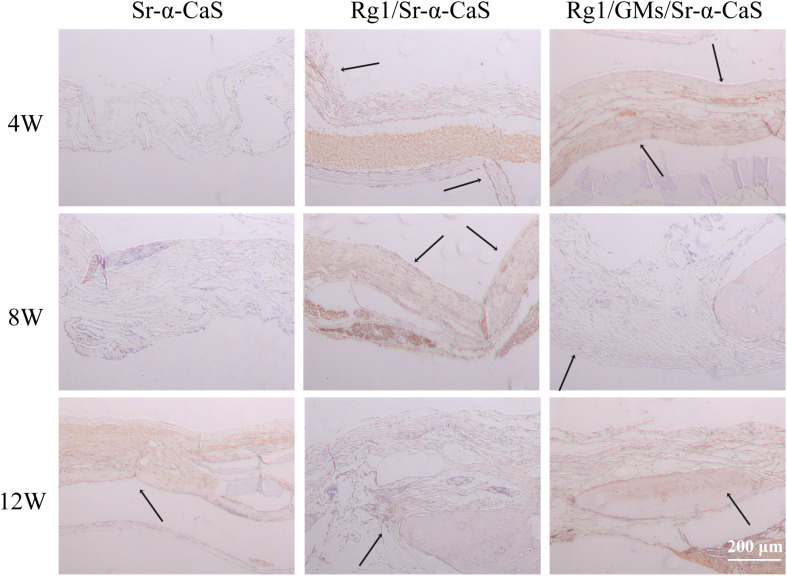
OCN expression of Sr-α-CaS, Rg1/Sr-α-CaS and Rg1/@GMS/Sr-α-CaS group (Magnification = 100×, black arrow: OCN).

Osteocalcin, a structural protein from osteoblast, played an important role in regulating bone calcium metabolism and was processed to produce carboxylate osteocalcin to participate bone development. In Figure 8 and Supplementary Figure S6C, every scaffold showed low expression of OCN at 4 week. Rg1 loaded scaffolds showed higher OCN expression (black arrows) at 8 weeks, which was more obvious at 12 weeks. Therefore, sustained release of Rg1 accelerated bone regeneration *in vivo*.

## Discussion

Biomaterials of Sr modified calcium sulfate hemihydrate have been reported in bone repair. The scaffold of Sr-CaS showed good biocompatibility and repair in tibia bone defect of SD rats, which activated TGF-β/Smad signaling pathway to improve bone formation (Liu et al., 2019). Traditional Chinese medicine recently has become a hot research topic in life sciences. As a precious medicine, Panax ginseng C. A. has always been regarded as the king of herbs. Ginsenosides are considered to be active ingredients in ginseng (Lee and Kim, 2014; Biswas et al., 2017; Mohanan et al., 2018). The dose of Ginsenosides Rg1 was reported to have influence on cell growth and tissue differentiation such as adipose-derived stromal cells (ASCs) (Liang et al., 2019), neural stem cells (Li et al., 2015; Gao et al., 2017a), and human dental pulp cells (hDPCs) (Wang et al., 2012). For the cartilage repair and regeneration, the dosage of Rg1 was related to cell proliferation and chondrogenic phenotype differentiation of breast adipose-derived stem cells HBASCs (Xu et al., 2015). The dose-effect of Rg1 was also found in human periodontal ligament stem cells (hPDLSCs), and concentration from 10 to 100 μmol/L was investigated. Rg1 at 10 μmol/L significantly enhanced the proliferation and osteogenic differentiation of hPDLSCs while 100 μmol/L Rg1 caused cytotoxicity (Yin et al., 2015).

In this study, we firstly introduced Rg1 to Sr-α-CaS scaffold, and both of Rg1 and Sr accelerated repair and regeneration of cranial bone. To avoid drug burst release, Rg1 was loaded to gelatin microspheres. From the results of Micro-CT in Rg1/GMs/Sr-α-CaS group, the new bone volume was about 83.3% in 12 weeks and BMD increased to 1133 mg/cm^3^. Scaffolds also need to provide mechanical support and the mechanical properties should be as close as possible to normal tissue. As we knew, single calcium sulfate hemihydrate scaffolds were absorbed and metabolized too quickly for its rapid degradation. With the incorporation of Sr and Rg1/GMs, mechanical properties were improved and the Rg1 release was prolonged, which made scaffold of Rg1/GMs/Sr-α-CaS a promising candidate for bone repair. The main components of natural bone are hydroxyapatite and collagen. In order to match the degradation rate and mechanical properties with the natural bone, we are considering to prepare composite scaffolds with cross-linked natural biomaterials. To mimic the structure and function of natural bone tissue, natural biomaterials such as collagen, silk fibroin, and chitosan have been reported to add to calcium sulfate hemihydrate (Liu et al., 2015a, 2018; Chen et al., 2016).

Furthermore, dose of Rg1 also influenced the osteogenic differentiation and angiogenesis of MC3T3-E1 *in vitro*. In Figures 3A,B, Rg1/GMs/Sr-α-CaS group always showed higher expression of ALP and VEGF than Rg1/Sr-α-CaS. However, due to the degradation of scaffolds and sustained release of gelatin microspheres, the amount of Rg1 in leaching solution of Rg1/Sr-α-CaS was more than Rg1/GMs/Sr-α-CaS. The low concentration of Rg1 accelerated the osteogenic differentiation and vascularization of MC3T3-E1 *in vitro* while it was not obvious at high concentration. In future research, we would explore the effect of Rg1 concentration on cell proliferation and differentiation *in vitro* and tissue repair and regeneration *in vivo*.

In summary, this study prepared a novel Rg1/GMs/Sr-α-CaS scaffold to repair and regenerate cranial bone tissue. The scaffolds showed good biocompatibility and promoted osteogenic differentiation and vascularization *in vitro* and *in vivo*. The Rg1/GMs/Sr-α-CaS scaffolds combined traditional Chinese medicine with modern science and technology, and provided a possible way to study traditional herbs and a potential approach for bone repair and regeneration in clinic.

## Data Availability Statement

All datasets presented in this study are included in the article/Supplementary Material.

## Ethics Statement

All experimental protocols were approved by the Ethics Committee in the Nanshan Hospital (protocol # 2018-C-106).

## Author Contributions

DY and ST designed the experiment. PL and QL completed the experiment and obtained the data. LY and CW also made substantial contributions to the experiment including the study design, *in vitro* and *in vivo* study, the acquisition, analysis, and interpretation of data for the work. All authors contributed to the article and approved the submitted version.

## Conflict of Interest

The authors declare that the research was conducted in the absence of any commercial or financial relationships that could be construed as a potential conflict of interest.
