# Supplementary material for: Strontium Modified Calcium Sulfate Hemihydrate Scaffold Incorporating Ginsenoside Rg1/Gelatin Microspheres for Bone Regeneration
Source: Front Bioeng Biotechnol. 2020 Aug 18;8:888. doi: 10.3389/fbioe.2020.00888 (PMC7461947; doi:10.3389/fbioe.2020.00888)
Supplement: Supplementary file 1 [file Image_1.pdf]

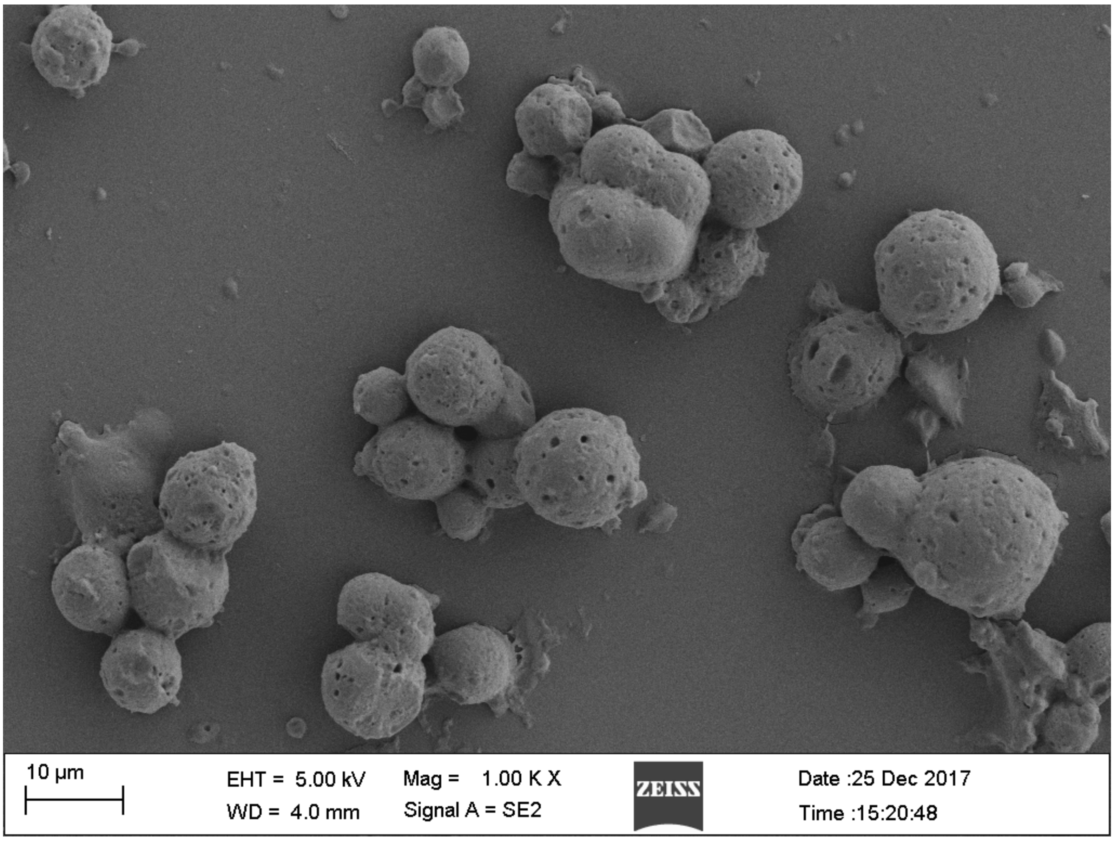

Fig.S1 The SEM image of gelatin microspheres.

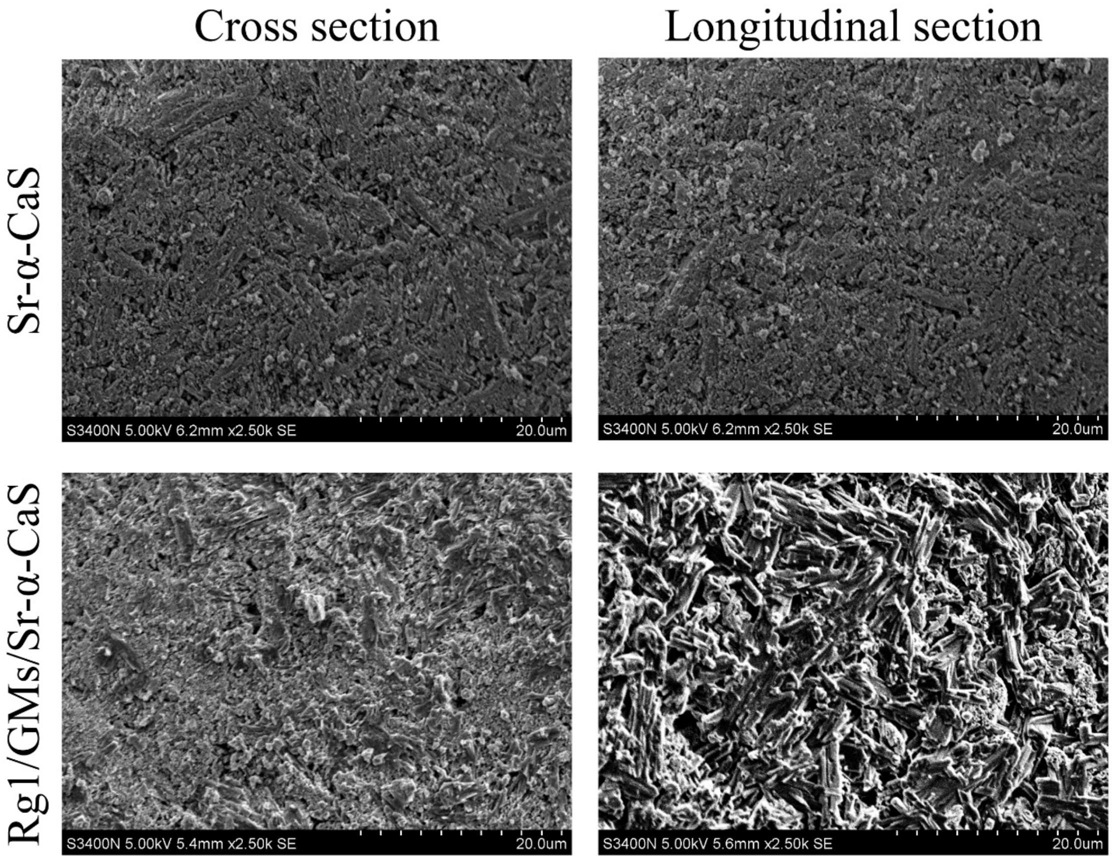

Fig. S2 The SEM image of Sr-α-CaS and Rg1/GMs/ Sr-α-CaS scaffolds.

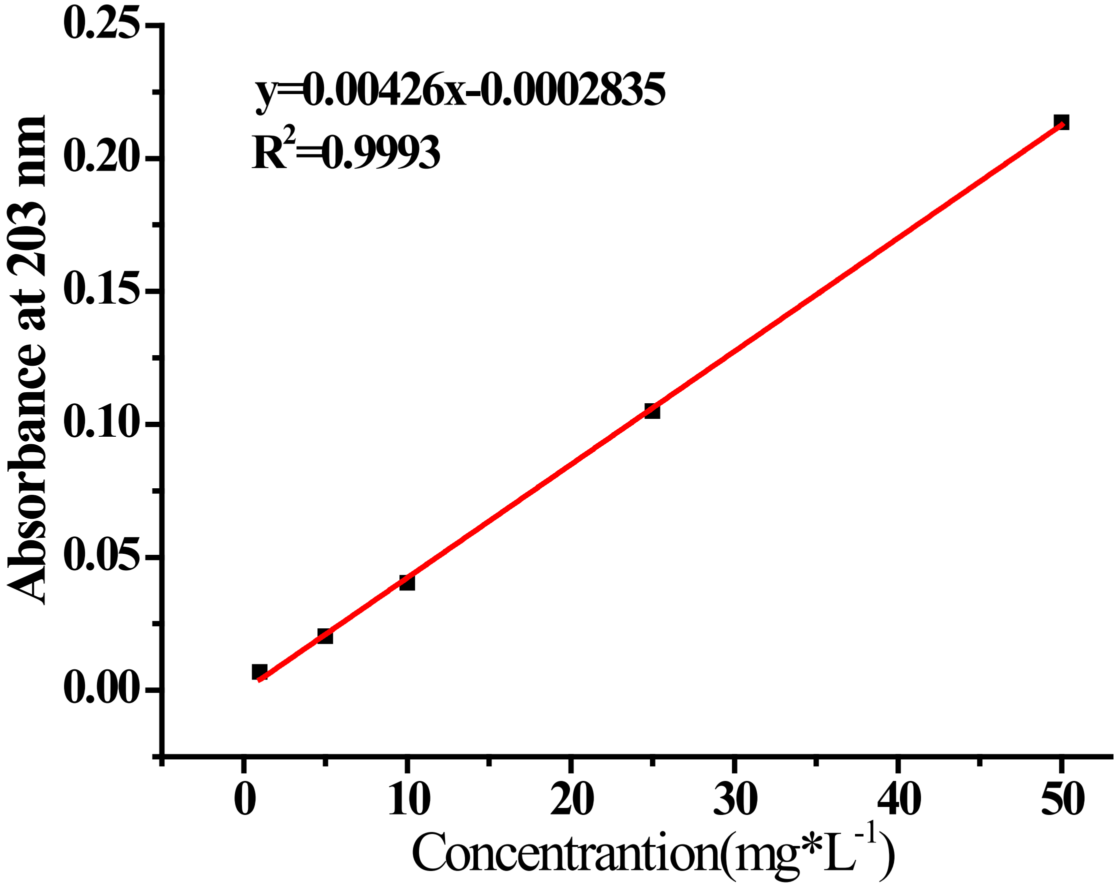

Fig. S3 The standard curve of Ginsenoside Rg1.

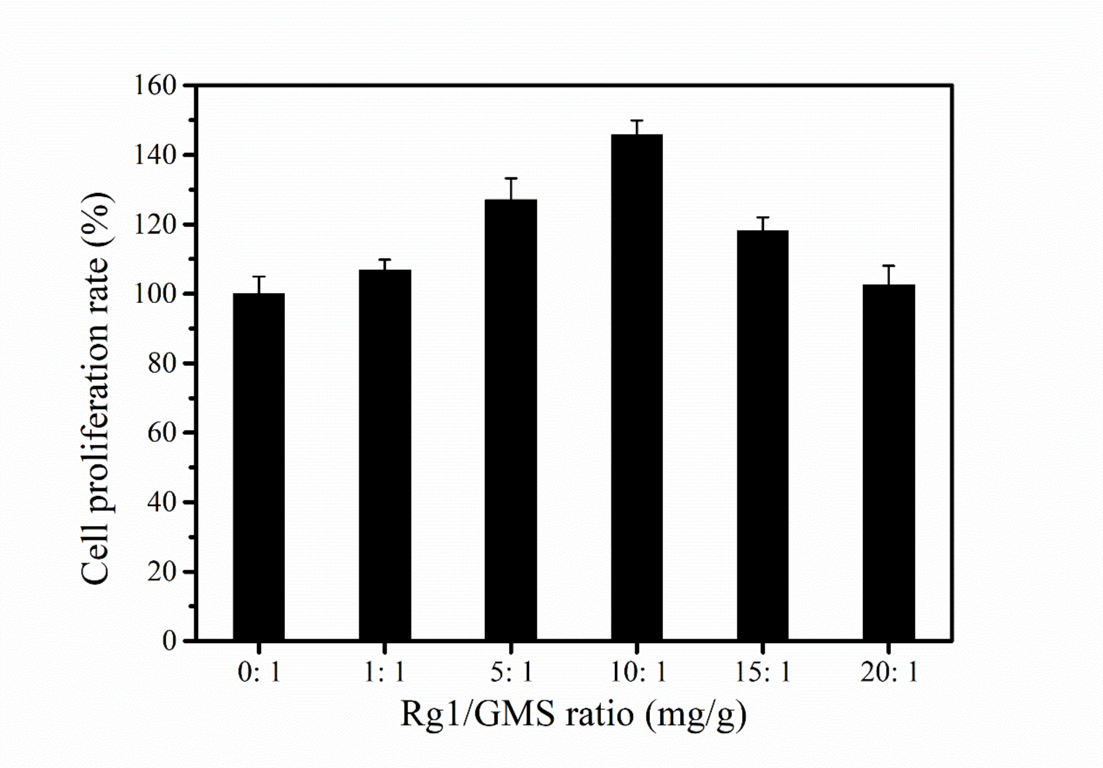

Fig. S4 Cell proliferation rate under different ratio of Rg1/GMs.

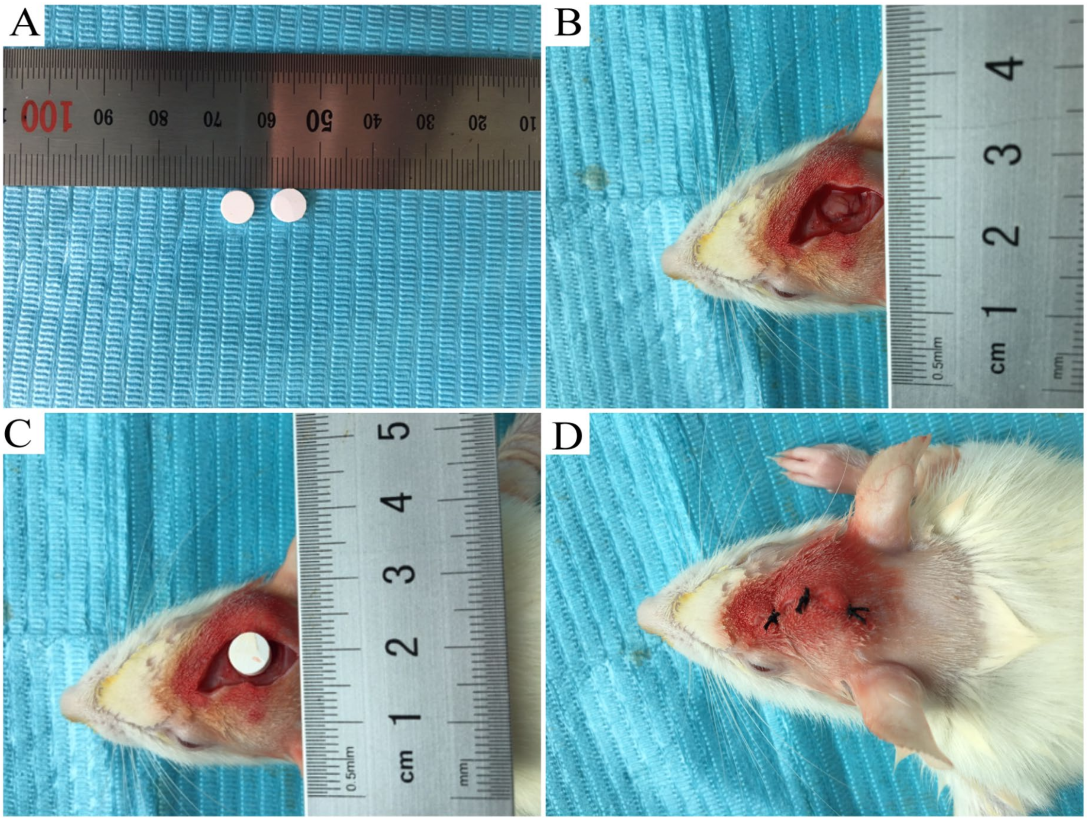

Fig.S5 Surgical process of composite scaffolds transplantation into rat calvarial defects.

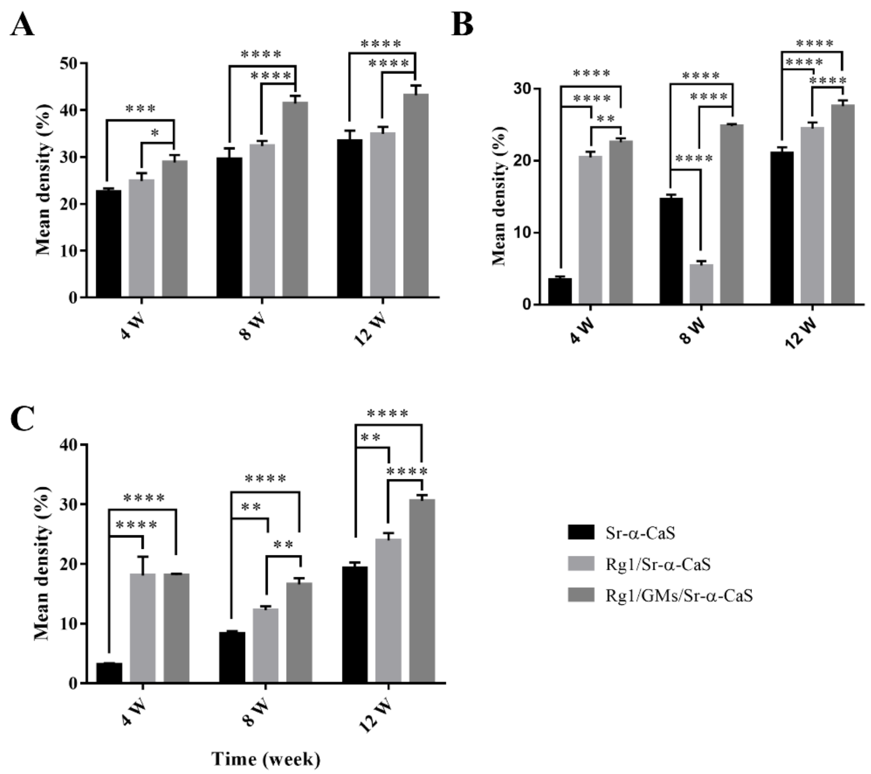

Fig.S6 Statistical analysis of Masson staining (A), SafraninO-staining (B) and Immunohistochemistry (C)
